# Progress in the Molecular Biology of Ewing Tumors

**DOI:** 10.1080/13577149878109

**Published:** 1998-03

**Authors:** Heinrich Kovar

**Affiliations:** Children's Cancer Research Institute (CCRI) St Anna Kinderspital Kinderspitalgasse 6 Vienna A-1090 Austria

## Abstract

*Purpose/results/discussion.* Rearrangement of the *EWS* gene with an *ETS* oncogene by chromosomal translocation is a hallmark of the Ewing family of tumors (EFT). Detectability, incidence, tumor specificity and variability of this aberration have been matters of intense investigation in recent years. A number of related alterations have also been found in other malignancies. The common consequence of these gene rearrangements is the generation of an aberrant transcription factor. In EFT, the ETS partner is responsible for target recognition. However, synergistic and possibly tissue-restricted transcription factors interacting with either the EWS or the ETS portion may influence target selection. Minimal domains of both fusion partners were defined that have proved necessary for the *in vitro* transformation of murine fibroblasts. These functional studies suggest a role for aberrant transcriptional regulation of transforming target genes by the chimeric transcription factors. Also, fusion of the two unrelated protein domains may affect overall protein conformation and consequently DNA binding specificity. Recent evidence suggests that EWS, when fused to a transcription factor, interacts with different partners than germ-line EWS. Variability in EWS–ETS gene fusions has recently been demonstrated to correlate with clinical outcome. This finding may reflect functional differences of the individual chimeric transcription factors. Alternatively, type and availability of specific recombinases at different time-points of stem cell development or in different stem cell lineages may determine fusion type. Studies on EFT cell lines using EWS–ETS antagonists do suggest a rate-limiting essential role for the gene rearrangement in the self-renewal capacity of EFT cells. The presence of additional aberrations varying in number and type that may account for immortalization and full transformation is postulated. Knowledge about such secondary alterations may provide valuable prognostic markers that could be used for treatment stratification.


*Sarcoma* (1998) **2**, 3–17

REVIEW

### Progress in the molecular biology of Ewing tumors

HEINRICH KOVAR

*Children's Cancer Research Institute, Vienna, Austria*

**Abstract**

*Purpose/results/discussion.* Rearrangement of the *EWS* gene with an *ETS* oncogene by chromosomal translocation is a hallmark of the Ewing family of tumors (EFT). Detectability, incidence, tumor specificity and variability of this aberration have been matters of intense investigation in recent years. A number of related alterations have also been found in other malignancies. The common consequence of these gene rearrangements is the generation of an aberrant transcription factor. In EFT, the ETS partner is responsible for target recognition. However, synergistic and possibly tissue-restricted transcription factors interacting with either the EWS or the ETS portion may influence target selection. Minimal domains of both fusion partners were defined that have proved necessary for the *in vitro* transformation of murine fibroblasts. These functional studies suggest a role for aberrant transcriptional regulation of transforming target genes by the chimeric transcription factors. Also, fusion of the two unrelated protein domains may affect overall protein conformation and consequently DNA binding specificity. Recent evidence suggests that EWS, when fused to a transcription factor, interacts with different partners than germ-line EWS. Variability in EWS–ETS gene fusions has recently been demonstrated to correlate with clinical outcome. This finding may reflect functional differences of the individual chimeric transcription factors. Alternatively, type and availability of specific recombinases at different time-points of stem cell development or in different stem cell lineages may determine fusion type. Studies on EFT cell lines using EWS–ETS antagonists do suggest a rate-limiting essential role for the gene rearrangement in the self-renewal capacity of EFT cells. The presence of additional aberrations varying in number and type that may account for immortalization and full transformation is postulated. Knowledge about such secondary alterations may provide valuable prognostic markers that could be used for treatment stratification.

**Key words**: EWS, ETS, IGF1, *tumor suppressor, prognosis.*

### Introduction

Ewing's sarcoma (ES), being a rare malignant disease affecting bone and soft tissue in children and young adults, was hardly known to people other than pediatric oncologists until the characterization of a chimeric gene product presumed to be causally involved in the generation of this neoplasm. It dramatically gained attention when, from investigating other malignancies, it became apparent that the ES-derived oncoprotein constitutes the prototype of a whole class of aberrant proteins specifically associated with certain tumor types. Consequently, ES may be considered a model system to study malignant conversion on a subclinical level. The discovery of the ES-associated gene rearrangement transiently halted a controversy among pathologists about the existence of distinct categories of ES (i.e. osseous ES, extra-skeletal ES, Askin tumor, peripheral primitive neuroectodermal tumor) because it was found to be expressed in all of them. Clinically, however, there is a need for diversification. Although more than half of the patients can be cured by multimodal therapy, one third of cases with localized disease and about 80% of patients presenting with metastases succumb to the disease (for a recent review, see Kovar *et al.*[1]). Current treatment protocols have largely compensated for classical prognostic markers such as tumor volume and localization of the primary except for the rather unfavorable presence of metastases at diagnosis. It is likely, therefore, that biological differences exist between so far incurable aggressive disease and clinically manageable localized disease inexplicable by the mere presence of the ES-associated gene rearrangement. While Ewing's tumor research has focused on the clinical exploitability and the function of the ES-specific gene rearrangement since its discovery in 1992, this review will also consider extensively the role of additional molecular aberrations in the search for useful prognostic markers. Neoplastic transformation and metastatic spread is commonly believed to result from a multi-step pro-

Correspondence to: H. Kovar, Children's Cancer Research Institute (CCRI), St Anna Kinderspital, Kinderspitalgasse 6, A-1090 Vienna, Austria. Tel: +43 1 40470 ext, 409; Fax: +43 1 4087230; E-mail: kovar@ccri.univie.ac.at.

1357-714X/98/040003-15 ©1998 Carfax Publishing Ltd cess. In this context, the ES-specific gene rearrangement obviously constitutes a rate-limiting event. According to Knudson's legendary two-hit hypothesis, at least one additional aberration should be present in a Ewing tumor. It is possible that this second hit is less specific and affects different genes at different times during development of the enigmatic Ewing tumor stem cell, thus defining distinct subcategories of the disease. Consequently, Ewing tumor research is slowly moving towards molecular subclassification and staging.

### Diagnostic tools

In 1988, the cytogenetic translocation t(11;22)(q24;q12) was described as specifically associated with histopathologically diagnosed ES and peripheral primitive neuroectodermal tumor (pPNET).[2] The presence of this aberration in a largely undifferentiated small round cell tumor of childhood turned out to be a formidable diagnostic marker.[3,4] However, cytogenetic analysis was restricted to tumor cells with at least limited *in vitro* proliferation potential. The generation of an antibody, HBA71,[5] specifically reacting with the surface glycoprotein encoded by the *MIC2* gene,[6] which was found to be abundantly present in tumor cells carrying a chromosome 22q12 aberration,[7,8] enlarged the spectrum of diagnostic tools. However, embryonal rhabdomyosarcomas, asterocytomas, neuroendocrine tumors and carcinomas occasionally stained positive with HBA71[9] and, when using a more sensitive antibody (12E7[10]), high level expression of this antigen was also noted in early hematopoietic precursor cells[11] and several lymphomas.[12] The characterization of the ES break-point regions on chromosomes 22 and 11[13] and the subsequent cloning of a chimeric cDNA resulting from a gene fusion between a novel gene, designated *EWS*, and the ETS transcription factor gene *FLI1*[14] allowed for sensitive detection of tumor cells carrying a 11;22 translocation even in small samples of fresh, frozen or paraffin-embedded material by means of reverse transcriptase polymerase chain reaction (RT-PCR).[15–18] Subsequently, several alternative fusion partners for *EWS* from the *ETS* oncogene family were identified in ES and pPNET cases[19–24] Using the RT-PCR method, combined with genomic analysis of the *EWS* break-point region, on a large series cf osseous and extra-skeletal ES (including Askin tumors and pPNET, designated Ewing family of tumors (EFT)) as opposed to several unrelated small round cell tumors, the specificity of the *EWS–ETS* gene rearrangement and the correlation with high MIC2 expression was confirmed.[16–25] Recently, however, the limitation of this aberration to typical EFT members has been questioned since RT-PCR amplifiable *EWS–FLI1* fusion transcripts have been reported in childhood soft tissue sarcomas with mixed phenotype,[26–27] in some olfactory neuroblastomas[28] which have previously been shown not to express MIC2,[29] and in two cases of classical MIC2-negative neuroblastoma.[30] In the absence of any cytogenetic evidence for a t(11;22) in neuroblastoma the latter finding needs to be independently confirmed. On the other hand, RT-PCR failed to demonstrate the presence of chimeric EWS transcripts in roughly 5% of histologically classified EFT. Sceptics might raise their fingers and recall all the potential pitfalls of using RT-PCR including the method's inherent susceptibility to cross-contamination as a single tool in the diagnosis of histopathologically ambiguous cases of small round cell tumors. Intriguing questions, i.e. if *EWS–ETS* gene rearrangements can occur outside the EFT and if 'atypical' ES exist, can only be assessed by the use of complementary techniques allowing for the visualization of the *EWS* gene rearrangement on a single cell level. It has already been demonstrated that fluorescent *in situ* hybridization (FISH) using cosmids flanking the EFT break-point regions is not restricted to metaphase chromosomes, but is also feasible to detect the gene rearrangement efficiently on interphase nuclei [31,32] (Hattinger *et al.*, unpublished). Alternatively, antibodies to unique domains of the chimeric gene product could allow the routine pathologist to screen for the *EWS* rearrangement by standard immunohistochemical methods. The author and others[125, 126] have recently obtained preliminary indirect evidence from protein interaction studies that an amino terminal EWS domain, which appears to be inaccessible in germ-line EWS, might be specifically exposed on the surface of the chimeric product (see below). One could endeavor, therefore, to generate an agent that distinctly recognizes the altered conformation of the EWS portion present in EWS fusion proteins. The hinge region of EWS–ETS chimeric proteins displays a high degree of variability due to variable break-point locations in the genes contributing to the translocation. So far, five alternative *ETS* family members have been found in *EWS* gene rearrangements. Therefore, antibodies to the linker domain of fused partners would be of only limited use in routine diagnosis. For the analysis of RT-PCR negative 'atypical' EFT and for small round cell tumors with a diagnosis other than EFT but RT-PCR positive for an EWS chimeric transcript, it is strongly recommended to confirm the molecular diagnosis by the demonstration of an EWS aberration on either DNA level (FISH or Southern blot) or on RNA level by Northern blotting. Presently, it cannot be excluded that using these approaches, followed by refined cloning procedures, further *ETS* family members will be identified as alternative fusion partners for *EWS* in EFT or non-EFT.

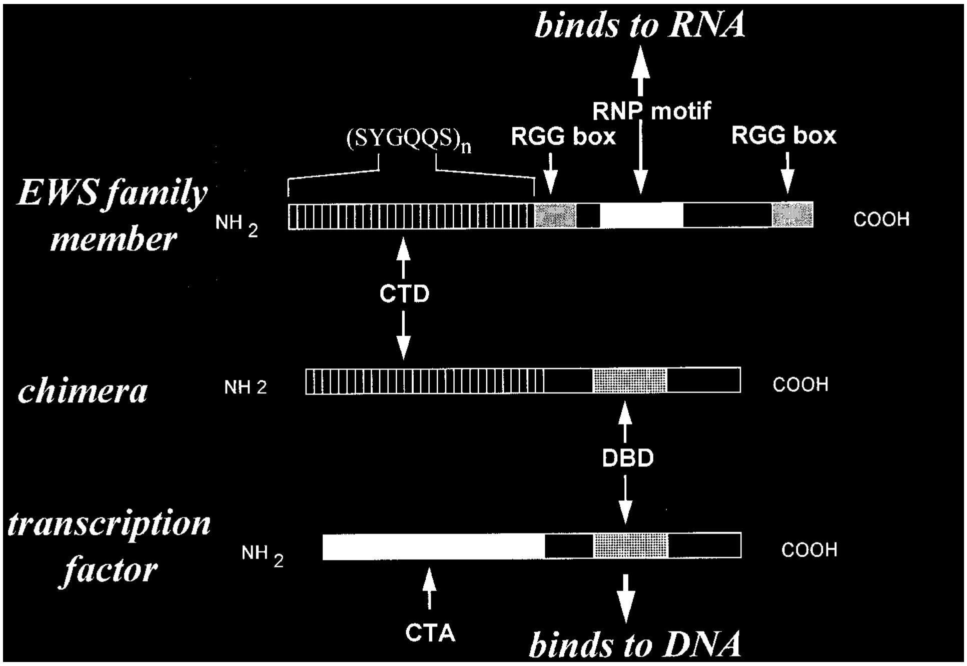

**Fig. 1.** *Generation of chimeric oncoproteins involving an EWS family member and a transcription factor. Protein domains presumably involved in RNA binding (RGG boxes and RNP motif) are replaced by the DNA binding portion of the transcription factor. The minimal domains of the fusion partners present in all chimeras are the carboxy terminal domain (CTD) of the EWS family member and the DNA binding domain (DBD) of the transcription factor. The carboxy terminal transactivation domain (CTA) of the transcription factor is lost in fusions of ETS family members but not of CHOP.*

### The *EWS–ETS* gene rearrangement

### *The* EWS *gene family*

EWS is the prototype of a growing family of putative RNA-binding proteins including TLS (translocated in liposarcoma)/FUS,[33–35] $hTAF_{II}68$ (TATA box binding protein associated factor),[36] the small nuclear ribonuclear protein(snRNP)-associated 69-kDa protein,[37] the bovine Pigpen protein[38] and *Drosophila cabeza*/SARFH (sarcoma associated RNA binding fly homologue),[39,40] that share distinct structural characteristics such as a conserved RNA binding motif flanked by arginine–glycine–glycine (RGG) boxes[41] and a putative zinc-finger domain in the carboxy terminus. This portion is replaced by the DNA binding domain of a transcription factor in the oncogenic EWS and TLS fusion proteins. The amino terminus is rich in glutamine and proline residues. As such, it resembles the activation domain of certain transcription factors such as SP-1.[42] In EWS, this *N*-terminal domain (NTD), which is encoded by the first seven exons,[43] is comprised of 30 copies of a repeated degenerate peptide of 7–12 residues rich in tyrosine, serine, threonine, glycine and glutamine[44] (Fig. 1). TLS was identified as a heterogenous nuclear ribonucleoprotein (hnRNP) in non-spliceosomal complexes on mRNA continuously shuttling between the nucleus and the cytoplasm[45] and SARFH was found to be associated with regions of the *Drosophila* chromatin transcribed by RNA polymerase II. Consistent with a role of EWS family members in gene transcription, $hTAF_{II}68$, TLS and EWS have been identified in subpopulations of the general transcription factor $TF_{II}D$.[126] However, recent evidence suggests that the oncogenic derivatives of TLS and EWS are not stably associated with the RNA polymerase II complex and $TF_{II}D$.[126] Accumulation in nuclear inclusions such as the coiled body and the nucleolus have been reported for Pigpen,[46] the 69-kDa snRNP-associated protein,[37] and, after transcriptional inhibition, for TLS.[47] Such nuclear subcompartments might either constitute the site of normal function of these EWS-related proteins or serve as their reservoir. Interestingly, oncoproteins that contain the amino terminal domain of EWS or TLS are also targeted to the same structure[47]. So far, the functional relevance of this finding is completely unknown.

### *A role for* EWS *and its partner genes in determining the EFT phenotype*

Figure 2 summarizes all known gene fusions involving either *EWS* or *TLS* in human malignancies. In an NIH3T3 transformation study, the type of transcription factor contributing to the chimeric gene product determined cell morphology.[35] This observation might in part explain why only members of the ETS transcription factor family are found in gene fusions with EWS associated with an EFT phenotype. However, while EWS and TLS amino termini appear to be functionally interchangable when fused to the transcription factor CHOP in the *in vitro* model,[35] as well as in myxoid chondrosarcoma,[33,34,48] TLS has never been found to replace EWS in EFT. In contrast, fusion of TLS to the ETS family member ERG, which is involved in 10% of EFT, has been reported for poor prognosis, t(16;21) positive, acute myeloid leukemia.[49–51] Rearrangement of *EWS* with other transcription factor genes such as *ATF1*, the Wilms' tumor gene *WT1* and the nuclear receptor *CHN/TEC* have been shown to be associated with malignant melanoma of soft parts, desmoplastic small round cell tumor and myxoid chondrosarcoma, respectively.[52–55] Thus, it is the specific combination of *EWS* with a subset of *ETS* transcription factor genes and/or a particular stem cell in which these genes are sensitive to illegitimate recombination that determine the EFT phenotype. Accessibility to rearrangement by an as yet undefined recombinase might also determine the incidence of EFT. Zucman *et al.* reported recently that sequence analysis of the entire *EWS* intron 6 region close to the major break-point region in EFT from Caucasian origin revealed a very high density of Alu elements resulting from repeated retroposition during evolution.[56] The Alu family of short interspersed repetitive DNA elements has previously been demonstrated to be frequently involved in human gene rearrangements.[57] This region was found to be reduced by 50% due to deletion in the African population. This inter-ethnic polymorphism in the *EWS* gene is accompanied by a striking difference in the incidence of EFT between populations of European and African origin.[58,59] It should be noted that the majority of *EWS* genomic break-points occur in intron 7 and that intron 6 is, in fact, never directly rearranged in EFT. So far, only three *EWS* genomic break-points have been sequenced, two in EFT and one in a desmoplastic small round cell tumor,[60–62], none of which contained Alu elements in the immediate vicinity of the rearrangement sites. Thus, direct proof for the involvement of Alu elements in *EWS* translocation is not available. In the published cases, the lack of uniformity of sequences affected by the gene rearrangement does not allow the identification of a specific recombinase responsible for the translocation. Chromosome 22 alteration may occur as the only cytogenetically visible aberration in EFT suggesting that the *EWS–ETS* gene rearrangement is not the consequence of a general genomic destabilization. However, the frequent involvement of more than two chromosomes in complex chromosome 22 aberrations and evidence for deletion of considerable amounts of sequences from the directly involved genes on the untranscribed counterpart of the derivative chromosome 22[60] imply a complex mechanism for gene rearrangement in EFT. In addition, while *EWS* and *FLI1* are equally oriented on the long arms of chromosomes 22 and 11 from the centromere to the telomere allowing for simple reciprocal translocation, the *ERG* gene is oriented in the opposite direction. Consequently, *EWS–ERG* gene fusions may result from either interstitial deletion/insertion mechanisms[63] or from complex genomic rearrangements involving additional chromosomes.[24] Although a high variability in *EWS* fusion partners and genomic break-point locations has been noted, rearrangement of *EWS* intron 7 with intron 5 or 4 of the *ETS* family gene *FLI1* predominates (about 80% of EFT cases).[16,18,24] Interestingly, the only known three cases of fusion between *EWS* and the *ETS* transcription factor gene *FEV* involve *EWS* intron 10 which is otherwise affected in only 9% of EFT. Since, as outlined later, the minimal portions of EWS and its fusion partners contained in all EFT-derived oncoproteins and required for full *in vitro* transformation and transcription activation function are significantly smaller than the portions present in the most frequently observed EWS fusions, it is unlikely that in *EFT* rearrangement sites are determined by functional constraints only. Rather, genomic structure and accessibility might direct illegitimate recombination to specific regions in the involved genes. Genomic accessibility and availability of recombinases might vary during the development of specific stem cell lineages. Therefore, it cannot be excluded that the different *EWS* rearrangements define different histogenetic starting points of EFT development. This model would provide an intriguing explanation for our recent observation of prognostic differences in EFT correlating with different *EWS* fusion types.[18] Alternatively, the various chimeric oncoproteins might display functional differences. Since the complete *EWS* and *FLI1* genes have been cloned and sequence information is readily available,[64–65] the description of more genomic rearrangement points in EFT will hopefully throw more light on the mechanism of gene rearrangement in this disease.

### *The* ETS *partner in the* EWS *fusion gene*

The ETS transcription factor family currently counts more than 30 members. It is characterized by the presence of a unique DNA binding domain which is highly conserved from flies to humans and has been first described for the viral oncogene v-*ets* of avian erythroblastosis virus E26 (E twentysix specific). Several *ETS* subfamilies can be defined on the basis of evolutionary sequence conservation. In ET, 95% of cases show *EWS* fusion to the *FLI1* (Friend leukemia virus integration site 1)/*ERG* (ETS related gene) subfamily of transcriptional activators.[16,18] These two gene products share, in addition to almost identical DNA binding domains, a 16-amino acid stretch immediately upstream of the ETS domain which is 100% conserved between *Xenopus* and humans, suggesting an important but as yet unidentified functional role.[66] This portion is retained in almost all EWS–FLI1 and EWS–ERG

| *EWS family member* | *transcription factor* (type) | *neoplasm* |
|---|---|---|
| EWS | FLI1 (ETS) | Ewing tumor (85%) |
| EWS | ERG (ETS) | Ewing tumor (10%) |
| EWS | ETV1 (ETS) | Ewing tumor (<1%) |
| EWS | E1AF (ETS) | Ewing tumor (<1%) |
| EWS | FEV (ETS) | Ewing tumor (<1%) |
| EWS | CHOP (bZIP) | *myxoid liposarcoma* |
| EWS | ATF1 (bZIP) | *melanoma of soft parts* |
| EWS | WT1 | *desmoplastic small round cell tumor* |
| EWS | TEC (SteroidR) | *myxoid chondrosarcoma* |
| TLS | CHOP (bZIP) | *myxoid liposarcoma* |
| TLS | ERG (ETS) | *acute myeloid leukemia* |
| TAF68 | ? | ? |
| 69kD | ? | ? |

**Fig. 2.** *Tumor-specific rearrangements between an* EWS *family gene and a transcription factor gene.*

fusions, while the genuine FLI1 and ERG transactivation domain is always replaced by the EWS amino terminus resulting in a potentiation of transcriptional activation properties.[67–69] The 85-amino acid DNA binding domain folds into three helices and a four-stranded ß sheet (winged helix-turn-helix motif),[70–72] most frequently refered to as 'the ETS domain'. In some ETS family members, this domain is flanked by auto-inhibitory $\alpha$ helical structures that fold back and interact with the ETS domain.[73] Structural studies on murine ETS1 suggest that upon specific binding to DNA a conformational change takes place that might expose distinct portions of the ETS domain and its flanking regions for interactions with other proteins.[74] DNA binding-dependent complex formation with other transcription factors mediated by the ETS domain and additional residues has been reported for several ETS family members including GABP$\alpha$, ELK1, SAP1, Pu1, ETS1 and ETS2.[75] As demonstrated for ETS1, when binding to the specific recognition sequence, intercalation of a tryptophan into the minor groove induces a sharp kink and a widening of DNA that might facilitate synergistic binding of other regulatory proteins.[76] Since almost all ETS proteins bind to a (G/C)(A/C)GGA(A/T)T consensus motif,[75,77] synergy with other transcription factors might determine target specificity of the individual ETS family members. Interestingly, ETS1, GABP$\alpha$, and FLI1 have recently been demonstrated to bind to the transcription factor PAX5 *in vitro*.[78] A flanking PAX5 binding site allowed for ETS1 binding to an imperfect ETS recognition motif indicating that cooperativity might change ETS binding specificity to some extent. Some ETS proteins, including ETS1, ETS2, FLI1, ERG, GABP$\alpha$ and TEL, share a further conserved region flanking the amino terminal transactivation domain, refered to as the 'pointed or B-domain'. This structure has been shown to define a specific oligomerization interface. For TEL, it governs homotypic aggregation.[79] No interaction partner has been defined for FLI1 and ERG so far. The conserved fold of the amino terminal domain of ETS proteins is, however, likely to underlie a conserved function. Thus, replacement of this portion by the EWS amino terminus may alter not only quantitatively but also qualitatively the transcriptional activation properties of these two ETS family members by placing them into a different protein context. In addition, several ETS family members have been demonstrated to be a target for the RAS/RAF MAP kinase signaling pathway. For ETS1, *ras* regulation involves phosphorylation of residues within the amino terminal 'pointed' domain. So far, no link between this signalling pathway and FLI1 or ERG has been reported. Consequently, it remains unclear if fusion to EWS will uncouple FLI1/ERG target gene regulation from extracellular signaling.

In about 1% of ET cases, *EWS* is rearranged with *FEV* (fifth Ewing's tumor variant) on chromosome 2.[21] This ETS family member displays 90% identity to FLI1 in the DNA binding domain but the FLI1- and ERG-specific flanking 16 amino acid sequence is missing in this protein. Interestingly, FEV also lacks an amino terminal transactivation domain present in most other ETS family members. Instead, it carries a long C-terminus which, because of the presence of abundant alanine residues, might serve as a putative repressor domain. However, experimental proof for such an activity is not yet available. Since this portion is retained in the EWS fusion, it remains to be established if the gene rearrangement would result in a functional conversion to an activator. Interestingly, germ-line *FEV* cDNA has been cloned from an *EWS–FLI1* expressing Ewing tumor cell line, indicating coexpression of the two genes within the same cell. If *EWS–FLI1* and FEV target the same genes and FEV operates as a repressor, it is possible that the EFT gene rearrangement results in the release of these genes from transcriptional inhibition by competitive binding.

So far, three cases of suspected EFT have been reported in which *EWS* was fused to ETS family genes of a different subclass on chromosomes 7p22 and 17q21, *ETV1* (ETS translocation variant 1) and *E1AF* (Adeno virus E1A enhancer binding factor), the putative human homologues of mouse *ER81* and

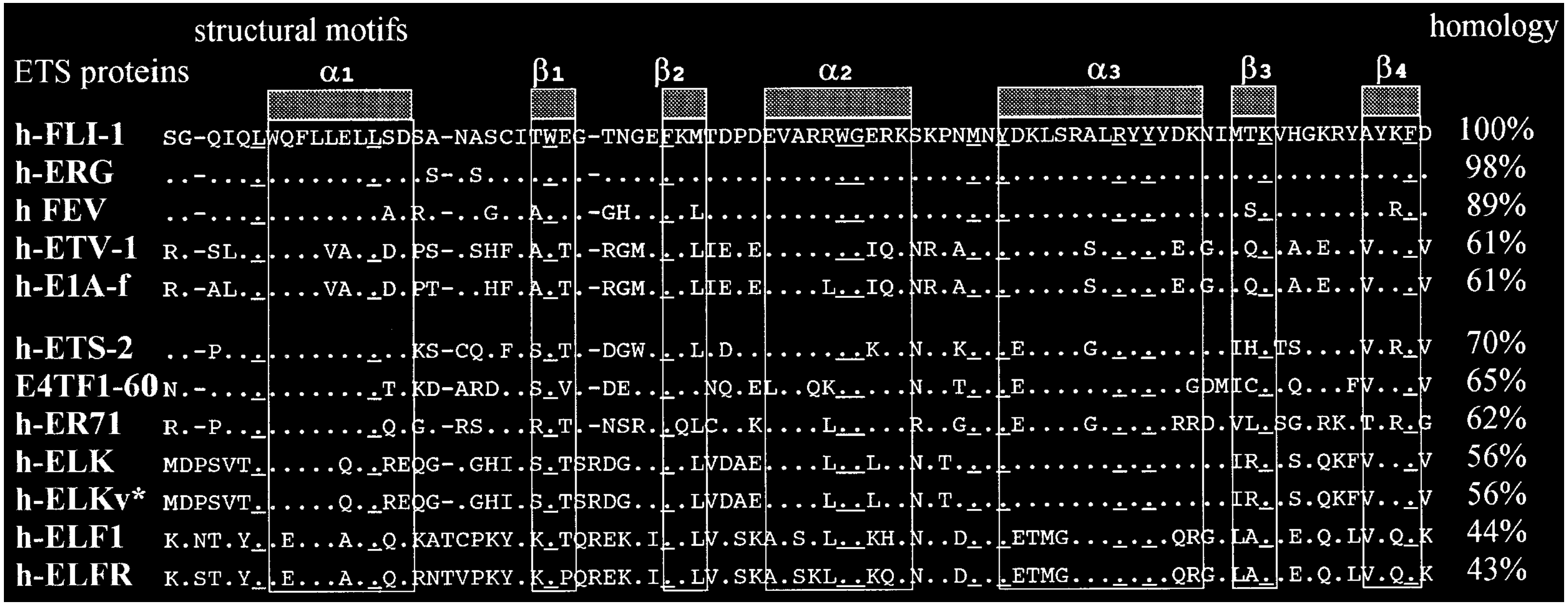

**Fig. 3.** *Comparison of DNA-binding domains of ETS proteins rearranged or coexpressed in Ewing's tumors. *: detectable only by RT-PCR; ●:identical amino acids; underlined: positions homologous to amino acids making contact to DNA in the PU.1 ETS-family member.*

*PEA3*, respectively.[19,20,23] Most notably, the DNA binding domains of these two transcription factors diverge from FLI1, ERG and FEV by 38% including the third $\alpha$-helix that contacts the central core of ETS binding sequences. Do EWS–ETV1 and EWS–E1AF chimeric transcription factors target the same genes as EWS–FLI1, EWS–ERG and EWS–FEV? By a subtractive cloning strategy for genes differentially expressed in EWS–FLI1-transformed and FLI1-transfected untransformed murine fibroblasts (NIH3T3), several potential EWS–FLI1-specific target genes were identified.[80] Among them were the murine homologue of cytochrome P-450 F1, cytokeratine 15, a novel SH2 domain containing protein, EAT2 (EWS–FLI1 activated transcript 2)[81] and the stromelysin gene. Stromelysin is a matrix metalloproteinase involved in metastatic invasion. Previously, E1AF has been demonstrated to regulate the stromelysin gene. In transfection experiments, E1AF was sufficient to confer an invasive phenotype to non-metastatic human breast cancer cells (MCF7)[82] and antisense RNA to E1AF was able to revert it in a squamous cell carcinoma cell line.[83] Stromelysin has also been demonstrated to be activated by ETS2, a member of another ETS subfamily.[84] However, coexpression of ERG, which also strongly binds to the same promoter but is by itself unable to activate it, resulted in inhibition of ETS2-mediated stromelysin gene activation.[85] Thus, different ETS family members, irrespective of their subclass, appear to compete for binding to specific target genes. It is likely, therefore, that target selectivity and the specific mode of gene regulation by the EWS chimeric ETS transcription factors strongly depends on the cellular background. A spectrum of ETS-related gene products coexpressed with EWS–FLI1 in EFT cell lines by amplification of ETS DNA binding domain encoding cDNAs has recently been defined using degenerate primers.[86] Among them, ETS2, E4TF1-60, ELK, ELF1, the putative human homologue of ER71, and a novel gene product ELFR were identified.[127] None of the ETS family members involved in EFT-specific gene rearrangements were found to be expressed in their germ-line configuration. This assay, however, may have missed low level expression of some ETS family members (i.e. FEV). As shown in Fig. 3 comparing the DNA binding domains of the ETS gene products alternatively fused to EWS in EFT with those found to be coexpressed with the chimeric transcription factors, ELK is the most likely ETS-related gene product that might interfere with the EFT fusion proteins in target site selection because the DNA-contacting third $\alpha$-helix of the ETS domain is identical to that of FLI1, ERG and FEV. ELK is one of several alternative ternary complex factors regulating a number of growth factor inducible genes.[87] In fact, EWS–FLI1 can replace ELK within the ternary complex formed on the serum response element of the *cfos* and the *EGR1* promoters.[88,89] In contrast to germ-line FLI1, binding of EWS–FLI1 to the serum response element did not require interaction with the serum response factor SRF. Ternary complex formation by FLI1 and EWS–FLI1 was mediated by a domain preceding the DNA binding domain and present in the majority of EFT-derived EWS–FLI1 fusions that show limited similarity to the ELK1–SRF interaction domain. However, no homologous structure can be identified in ERG and evidence for EWS–ERG involvement in ternary complex formation is not available.

In summary, current knowledge about normal and aberrant ETS proteins suggest a number of interesting candidate target genes for the EFT-specific chimeric transcription factors, potentially involved in the regulation of cell growth, signaling and metastasis, as revealed by the study of heterologous cellular systems such as murine fibroblasts. However, ample evidence exists that ETS transcription factor action is largely context specific. For EFT, the cell of origin remains a matter of speculation. Because of limited neural differentiation potential, EFT is considered as derived from the neuroectoderm.[90] Interestingly, *Xenopus FLI1* has been shown to be expressed in a restrictive pattern during embryogenesis evocative of neural crest cell invasion.[66] It is therefore speculated that *FLI1* might be involved in neural differentiation in the context of these early stem cells. If so, unscheduled activation of FLI-responsive genes by the EFT-specific EWS fusion proteins in an undifferentiated cell possibly unrelated to the neural crest might result in limited neural differentiation of the EFT stem cell depending on the degree of determination achieved at the time of gene rearrangement.[1] This model could explain the variable degree of neuroectodermal marker expression in ES and pPNET as well as the occurrence of biphenotypic tumors.[26]

### *The rate-limiting (first) hit in EFT pathogenesis*

*EWS–ETS* gene rearrangements are the only genetic aberrations that have so far been identified as highly associated with histologically diagnosed EFT. This association is the only available compelling argument that *EWS–ETS* gene rearrangements can be rate-limiting for tumorigenesis. Although complementary experimental evidence supports this assumption, the mechanism of malignant transformation by these chimeric oncoproteins remains elusive. The best studied biological model for the pathogenic role of inappropriately activated *FLI1* is Friend murine leukemia virus(F-MuLV)-induced erythroleukemia. Insertional activation of the *FLI1* gene appears to be the first detectable genetic change associated with this disease. The association between the detection of *FLI1* rearrangement and clonal outgrowth of erythroleukemia cells suggests that the activation of this transcription factor may be affecting the self-renewal potential of the infected erythroid progenitors. However, leukemogenesis proceeds in multiple steps and additional aberrations affecting viability of cells (e.g. inactivation of the tumor suppressor gene p53) can be observed in F-MuLV induced erythroleukemia.[91,92] In contrast to ERG,[93] normal FLI1 was reported to be unable to transform murine fibroblasts (NIH3T3) while expression of an EWS fusion protein resulted in pronounced anchorage-independent clonogenicity of NIH3T3 cells.[94,95] However, rat embryo fibroblasts and some murine fibroblast subclones were resistant to EWS–FLI1-mediated transformation. These findings again suggest that the oncogenic potential of normal and aberrant FLI/ERG ETS subfamily members may depend on a cell type-specific availability of relevant synergistic factors and possibly on the presence of additional aberrations. NIH3T3 transfection studies with various recombinant EWS–FLI1 deletion mutants revealed a dependence of transformation on both the EWS portion and the ETS domain.[95] However, optimal transactivation potential mediated by the 30 EWS amino terminal degenerate repeats included in almost all EFT-derived fusion proteins was dispensable for maximal focus formation of transfected NIH3T3 in soft agar. The minimal EWS domain required to transform murine fibroblasts was delineated to the first 82 amino acids.[94] Recently, evidence obtained shows that within the EWS–FLI1 fusion protein, but not within germ-line EWS, this peptide directly contacts a component of the RNA polymerase II complex, RPB7, and that this interaction is sufficient to drive EWS–FLI1-mediated reporter gene transactivation.[125] Interactions of full-length EWS with the general transcription factor TFIID, an essential component of the transcriptional preinitiation complex, were absent from EWS fusion proteins.[126] It is therefore possible that fusion of the EWS amino terminus to the FLI1 DNA binding domain alters the protein conformation and directly recruits RNA polymerase II to FLI1 target genes. Since RPB7 displays similarities to prokaryotic sigma factors, it might be involved in EFT-specific target site selection. Further protein–protein interactions presumably occurring downstream of the 82 amino acids might be required for efficient gene regulation within the EFT context. In addition, using the yeast two-hybrid protein interaction trap, further candidate proteins not directly related to transcription regulation were identified that interacted with the 82 amino acids long minimal transformation domain. These interactions await detailed characterization.

Since no tissue of EFT origin has been identified so far, transformation studies of authentic EFT stem cells cannot be performed. Alternatively, several investigators have used EWS–FLI1 antagonists (antisense RNA expression vectors, antisense oligonucleotides, dominant negative proteins) to modulate expression of the chimeric oncoprotein in EFT cell lines.[86,96–98] These studies revealed a growth inhibitory and anti-tumorigenic effect of these agents. Reduction in cell growth appeared to result from cell cycle arrest and not from reduced tumor cell viability. Recently, EWS–FLI1-mediated transformation of murine fibroblasts was demonstrated to require the presence of a functional insulin-like growth factor-1 (IGF1) receptor.[99] Interestingly, consistent expression of IGF1 and its receptor was previously reported for EFT and IGF1 was demonstrated to act as a potent growth factor for EFT cells in the absence of serum.[100–103] This cytokine appears to regulate negatively several mechanisms of programmed cell death at a far downstream step.[104] It has been shown that inhibition of the IGF1 autoregulatory circuit by anti-IGF1 receptor antibodies resulted in increased apoptosis and reduced tumorigenicity of EFT cells.[103] Taken together, the *EWS–ETS* gene rearrangement appears to be involved in the aberrant self-renewal capacity of EFT cells but might not be sufficient to guarantee survival of initiated tumor cells. However, as demonstrated recently, there might still be some role for FLI1, ERG and their EWS fusions to play in the protection from stress (i.e. calcium ionophore and serum deprivation)-induced cell death.[105]

### The second hit

Assuming that the *EWS–ETS* gene rearrangement is able to initiate EFT pathogenesis but is not sufficient to generate malignant transformation, the presence of additional mutations must be postulated. These aberrations might not necessarily be tumor specific but may display inter-individual variation that could account for variations in EFT phenotype as well as in clinical behavior. Also, they may determine differentiation capacity, invasive potential and treatment resistance. Consequently, while from a clinical point of view the *EWS–ETS* gene rearrangement provides a valuable diagnostic tumor marker, knowledge about the nature of additional aberrations in EFT may assist subclassification and provide prognostic tools. Since studies on facultative genetic anomalies in EFT have been largely neglected since the discovery of the *EWS–ETS* gene rearrangements, enhanced efforts to define the multitude of additional aberrations are warranted for the benefit of patients.

### *Clues from cytogenetics*

In three independent reviews of cytogenetically informative EFT cases, non-random numerical and structural chromosomal aberrations were reported to occur with variable frequencies in addition to the tumor specific t(11;22)(q24;q12)[106–107] (Hattinger *et al.*, unpublished). These include trisomy 8 in about 50% of cases frequently coupled with trisomy 12 occurring in roughly 20%, and a derivative chromosome 16 as a result of an unbalanced t(1;16) in 18% of EFT. In rarer cases, other aneuploidies have been identified. Structural chromosome 1 aberrations that either result in gains of chromosome 1q21–22 or relative losses of the short arm of chromosome 1, a frequent alteration in neuroblastoma and other neuroectodermal tumors, have also been observed in EFT (Hattinger *et al.*, unpublished). Interestingly, this chromosomal region harbours a gene encoding a protein (p73) that is structurally and functionally related to the tumor suppressor p53, a transcription factor involved in the regulation of cell growth and apoptosis, and frequently inactivated during the progression of many tumors.[108,109] While research currently focuses on the role of p73 for neuroblastoma pathogenesis, its relevance for a subset of EFT is something that has to be explored.

In general, excluding chromosome 22q12 translocations, numerical chromosome changes are the most frequent cytogenetic findings in EFT. These are likely to affect gene dosage. However, no candidate genes that could promote *EWS–ETS*-initiated EFT pathogenesis when expressed at aberrant levels have been identified so far. Also, information on genes affected by the recurrent chromosome 16 and 1 structural alterations is not available yet. Frequently, these cytogenetic alterations occur in only a subpopulation of neoplastic cells within the tumor suggesting that they may be associated with late stages of tumor progression.

### *The role of non-specific cancer genes*

In the absence of recurrent candidate progression-associated genetic alterations identifiable in EFT by the means of cytogenetics, work has focused on the analysis of mutations generally associated with a broad range of human malignancies. Genes investigated during the last years include the oncogenes *ras*, *cmyc* and *MDM2*, the tumor suppressors *p53*, *p16*, and *Rb*, the metatasis-associated splice variants of the CD44 adhesion molecule, and the tumor-specific metastasis suppressor gene *nm23H1*.[110–113,127] None of the studied oncogenes was found to be altered by mutation or in expression although occasional low level amplification of *MDM2* has been reported in an independent study on a similar sized cohort of EFT patients.[111] Neither mutation nor differences in expression levels of the nucleotide diphosphate-kinase *nm23H1* were observed irrespective of the disease extension.[112] Only standard *CD44* expression was detectable in EFT.[127] In contrast, homozygous deletions of the *p16* tumor suppressor was identified in about one-third of primary EFT samples. This finding was surprising since chromosomal aberrations of band 9q21 containing the *p16* gene were not reported before suggesting a high-frequency of microdeletions. Expression studies on EFT cell lines suggested that the frequency of *p16* inactivation might be even higher since post-transcriptional gene silencing was observed in several cases.[113] p16 acts as an inhibitor of the cyclin D1/cyclin-dependent kinase 4 (CDK4) complex that inactivates the cell cycle inhibitor pRb by phosphorylation. Inactivation of p16 should compromise the G1 cell cycle checkpoint. Over-expression of either cyclin D1 or CDK4, or loss of pRb function, is believed to mediate a similar effect.[114] In fact, we observed frequent cyclin D1 over-expression as well as variable CDK4 abundancy in EFT cell lines and loss of *Rb* in one case. However, expression data from primary tumor material are not available yet. In addition, low level *CDK4* amplification was previously reported for two of 30 EFT samples.[111]

In virally induced malignancy, G1 check-point control is frequently compromised by concomittant inactivation of the pRb and p53 pathways. In addition, F-MuLV-induced erythroleukemia involves not only the activation of the *FLI1* oncogene as a rate-limiting step but also mutation of p53. The author and others have, therefore, investigated the status and expression of *p53* and related genes in EFT.[110,115] The frequency of *p53* mutations in primary tumors was found to be lower than 10% as opposed to a mean frequency of 40–60% in most human malignancies. In contrast, in about half of EFT cell lines, the *p53* gene was mutated and showed loss of heterozygosity. Comparison of *p53* gene status between cell lines and the respective primary tumors of origin, when available, demonstrated that the observed increase in mutation frequency was due to selection and was not acquired during *in vitro* expansion of tumor cells. This result suggested that *p53* mutation might release EFT cells from some *in vivo* growth or survival factor dependency. Transient transfection and over-expression of wildtype *p53* in cell lines with endogenous mutant or wildtype gene status demonstrated frequent but variable reduction in apoptotic responsiveness,[116] suggesting the presence of some as yet unidentified cell-protective mechanism in EFT cell lines. Preliminary expression analysis of members of the cell death regulatory *Bcl2* gene family did not reveal any significant variations between individual EFT cell lines (our unpublished observations). Previously, high levels and activity of poly(ADP-ribose) polymerase, a nuclear enzyme that participates in DNA replication, repair and the triggering of apoptosis induced by DNA strand breaks, have been reported for some EFT cell lines.[117] Sensitivity of EFT cell lines to DNA damaging agents (etoposide, actinomycin D, X-rays) varied considerably in a manner independent from p53 responsiveness and endogenous *p53* gene status suggesting that complete mutational or partial inhibition of the p53 apoptosis pathway in EFT cell lines plays a role different from radio- and chemosensitivity. However, the physiological signals that stimulate p53-dependent cell death have not been defined so far.

### *Molecular markers of prognosis*

As a result of variable break-point localization in the involved genes, *EWS–ETS* gene products vary considerably in size. Most fusions include *EWS* exons 1 to 7 (89%) and *FLI1* exons 6 to 9 (54%). *EWS/FLI1* exon 7/6 fusions (type 1) predominate independent of the disease extension (51%). In about one-third of EFT, *FLI1* exon 5 is included into the chimeric gene product, most frequently joined to *EWS* exon 7 (type 2) (27%). In rare cases, the chromosomal translocation results in the inclusion of *EWS* exons 9 (1%) or 9 plus 10 (10%) or *FLI1* exon 4 (1%). In about 3% of cases, FLI1 exon 6 or exon 6 plus 7 are missing from the gene fusion. This variability has prompted us to investigate a possible prognostic impact of the gene fusion type. The study, performed on 55 patients with localized disease and 30 patients with metastases at diagnosis, treated according to the European Intergroup Coordinated Ewing's Sarcoma Studies (CESS 86 and EICESS 92), revealed a significantly better outcome for patients with localized disease carrying a type 1 *EWS–FLI1* expressing tumor as compared to non-type 1 cases.[18] A recent update after a median observation time of $3\frac{1}{2}$ years confirmed this result (Zoubek *et al.*, unpublished). In addition, an independent American study performed on a similar sized cohort of patients after a median follow up of 31 months, using a similar treatment regimen, not only supported our findings but also identified the *EWS–ETS* gene fusion type as a prognostic marker independent from the presence of metastases at diagnosis in a multi-variate analysis.[128] About 55% of the 'non-type 1' group in the two studies were comprised of type 2 gene fusions. Because of the low incidence of the individual 'other-gene' fusion types, no distinction has been made between various non-type 1 subgroups so far. In the absence of a biological explanation for the observed prognostic differences, large collaborative prospective studies are warranted to highlight the specific chimeric molecules and the protein domains associated with adverse patients' outcome.

Still, about 20% of patients with localized tumors and more than half of the patients with metastases succumb from the disease despite the expression of a type 1 *EWS–FLI1* gene fusion, suggesting the existence of additional adverse factors.

Comparison of *p16* gene status and clinical course of 23 EFT patients analyzed so far suggested an adverse prognosis associated with this aberration independent from the extension of the disease.[113] These results, which have not been subjected to statistical analysis, must be considered as preliminary since patients' numbers in the study were small and the median observation period did not exceed 2 years. Retrospective immunohistochemical analysis of biopsy material from a large number of patients will help to clarify the prognostic relevance of a disrupted pRb cell cycle regulatory pathway in EFT. Also, mutation of *p53* might be linked to an adverse outcome, since none of the three EFT patients from our series carrying such an aberration survived. However, because of the rarity of this alteration it cannot serve as a useful prognostic marker.

A prognostic relevance for EFT of the observed numerical and structural cytogenetic changes has not been demonstrated with confidence due to low sample numbers in the studies performed so far. Most recently, deletion at 1p36, occurring in 6/22 localized EFT, was discussed as being associated with unfavorable outcome in this group (Hattinger *et al.*, unpublished).

In the absence of reliable molecular markers to predict outcome in EFT, the presence of clinically overt metastases at diagnosis is commonly considered as the only prognostic criterion that is used for treatment stratification. The *EWS–ETS* gene rearrangement as a tumor cell specific marker detectable by the highly sensitive RT-PCR method provides a powerful means for the detection of minute numbers of circulating tumor cells that may be the source of clinically occult micrometastases.[118,119] However, except shortly after surgical intervention,[120] mobilization of PCR detectable amounts of tumor cells ($>1/10^6$) into the bloodstream has rarely been observed. In contrast, tumor cells were detected at diagnosis by this method in the bone marrow of 30% of patients with localized disease, 50% of cases with isolated lung metastases and all patients with bone metastases.[120,121] In a preliminary series of 23 patients lacking clinically overt dissemination, RT-PCR screening for bone marrow involvement did not allow the prediction of early relapse after a median observation time of 30 months. It is, however, necessary to recall several factors that may affect tumor cell detection in the bone marrow by RT-PCR: (1) tumor cell infiltration may be focal and bone marrow aspiration may miss these sites, (2) bone marrow aspirates may contain variable amounts of diluting blood resulting in insufficient sensitivity, (3) primary EFT cells may differ in vitality, although diluted tumor cells from cell lines can be detected in blood samples even after 48 h at 4°C,[119] and (4) bone marrow infiltrating tumor cells may be in a resting state and express lower levels of chimeric *EWS* RNA than proliferating tumor cells. RT-PCR measures RNA quantity rather than tumor cell abundance. In a recent study, up to 10-fold variations in the content of chimeric *EWS–ETS* transcripts between individual EFT cell lines have been reported.[122] Moreover, germ-line *EWS* expression in T-cells has been demonstrated to depend on the proliferative activity.[123] Since the EFT-specific chromosomal rearrangement places the chimeric gene under the control of the *EWS* regulatory sequences, it is possible that *EWS–ETS* gene expression may also vary. Consequently, detectability of *EWS–ETS* chimeric transcripts does not necessarily reflect true tumor cell content. Even if RT-PCR studies fail to demonstrate significance of positive blood or bone marrow screening results for relapse in patients with localized disease, the question of prognostic relevance of tumor cell infiltration remains unsolved. To assess this problem, immunohistochemical studies may prove to be superior to RT-PCR. While MIC2 may serve as a valuable surface marker in tumor diagnosis, its exploitability for tumor cell detection in hematopoietic tissue is limited.[124] The recently discovered expression of gastrin-releasing peptide (GRP) by all EFT cell lines and about half of the primary tumors tested (Lawlor *et al.*, unpublished) may provide a marker that, in conjunction with MIC2, may allow the identification of EFT cells in blood and bone marrow with increased specificity. The study of *EWS–FLI1* transcriptional targets may result in the identification of tumor cell-restricted immunohistochemical markers. In order to detect positively staining cells with very low abundance on a routine basis, automated microscopic screening and consequently sophisticated technical equipment is warranted.

### Conclusions

In this review, I have summarized evidence for the importance of studying EFT-specific genetic alterations in an authentic cellular background. Since the histogenesis of EFT is still enigmatic and no experimental evidence for *EWS–FLI*-mediated tumorigenesis has been reported from transgenic mouse models so far, EFT cell lines remain the only available system for such investigations. In this institution, cell lines could be established from 12 EFT patients with well documented clinical course. If a cell line could be expanded from the primary tumor, all subsequent tumor samples also gave rise to a cell line. All but one patient died from the disease suggesting that establishing a cell line selects for patients with adverse prognosis. In fact, non-type 1 *EWS–ETS* gene fusions, *p16* deletions and *p53* mutations were clearly increased in EFT cell lines from 23 patients investigated. They may, therefore, represent the most therapy-resistant subpopulation of tumor cells despite variable *in vitro* sensitivity to cytotoxic agents. Thus, EFT cell lines may serve as a pool for the identification of putative bad prognostic markers. However, only large cooperative clinical studies and multivariate statistical analysis will help to address the question: how far can the identification of such markers translate into clinically useful criteria for treatment stratification? When comparing a wide spectrum of EFT-derived cell lines for marker expression and response to either differentiation inducing agents, growth factors or cytotoxic compounds, an immense variability was observed. Consequently, in order to sort out the biological defects common to all EFT, a large panel of genetically well defined cell lines will have to be investigated. In the long term, such studies will result in the identification of EWS–ETS-specific target genes and in a more detailed knowledge of the mechanism of malignant conversion of the enigmatic EFT precursor cell. Hopefully, for the benefit of the patients, this knowledge will potentially provide novel targets for therapeutic intervention.

### Acknowledgements

I thank Drs Enrique de Alava, Peter Ambros, Dave Aryee, Marc Ladanyi, Poul Sorensen, Laszlo Tora and Jeff Toretsky for permitting me to refer to their before publication results.

### References

1 Kovar H, Zoubek A, Gadner H. A long way from the definition of the molecular basis to benefit in the clinical management of Ewing tumours. *Onkologie* 1996; 19:234–40.
2 Turc Carel C, Aurias A, Mugneret F, *et al.* Chromosomes in Ewing's sarcoma. I. An evaluation of 85 cases of remarkable consistency of t(11;22)(q24;q12). *Cancer Genet Cytogenet* 1988; 32:229–38.
3 Aurias A [Cytogenetic data on sarcomas of the bone and soft tissue] Donnees cytogenetiques dans les sarcomes des os et des tissus mous. *Bull Cancer Paris* 1988; 75:423–9.
4 Turc Carel CS. [Contribution of cytogenetics to the diagnosis of Ewing's sarcoma and small round cell tumors] Apport de la cytogenetique au diagnostic du sarcome d'Ewing's et des tumeurs a petites cellules rondes. *Bull Cancer Paris* 1991; 78:77–84.
5 Hamilton G, Fellinger EJ, Schratter I, *et al.* Characterization of a human endocrine tissue and tumor-associated Ewing's sarcoma antigen. *CancerRes* 1988; 48:6127–31.
6 Banting GS, Pym B, Darling SM, *et al.* The MIC2 gene product: epitope mapping and structural prediction analysis define an integral membrane protein. *Mol Immunol* 1989; 26:181–8.
7 Kovar H, Dworzak M, Strehl S, *et al.* Overexpression of the pseudoautosomal gene MIC2 in Ewing's sarcoma and peripheral primitive neuroectodermal tumor. *Oncogene* 1990; 5:1067–70.
8 Ambros IM, Ambros PF, Strehl S, *et al.* MIC2 is a specific marker for Ewing's sarcoma and peripheral primitive neuroectodermal tumors Evidence for a common histogenesis of Ewing's sarcoma and peripheral primitive neuroectodermal tumors from MIC2 expression and specific chromosome aberration. *Cancer* 1991; 67:1886–93.
9 Fellinger EJ, Garin Chesa P, Triche TJ, *et al.* Immunohistochemical analysis of Ewing's sarcoma cell surface antigen p30/32MIC2. *Am JPathol* I991; 139:317–25.

10 Levy R, Dilley J, Fox RI, *et al.* A human thymus-leukemia antigen defined by hybridoma monoclonal antibodies. *Proc Natl Acad Sci USA* 1979; 76:6552–6.
11 Dworzak MN, Fritsch G, Buchinger P, *et al.* Flow cytometric assessment of human MIC2 expression in bone marrow, thymus, and peripheral blood. *Blood* 1994; 83:415–25.
12 Riopel M, Dickman PS, Link MP, *et al.* MIC 2 analysis in pediatric lymphomas and leukemias. *Hum Pathol* 1994; 25 :396–9.
13 Zucman J, Delattre O, Desmaze C, *et al.* Cloning and characterization of the Ewing's sarcoma and peripheral neuroepithelioma t(11;22) translocation break-points. *Genes Chromosomes Cancer* 1992; 5:271–7.
14 Delattre O, Zucman J, Plougastel B, *et al.* Gene fusion with an ETS DNA-binding domain caused by chromosome translocation in human tumours. *Nature* 1992; 359:162–5.
15 Adams V, Hany MA, Schmid M, *et al.* Detection of t(11;22)(q24;q12) translocation break-point in paraffin-embedded tissue of the Ewing's sarcoma family by nested reverse transcription-polymerase chain reaction. *Diagn Mol Pathol* 1996; 5:107–13.
16 Delattre O, Zucman J, Melot T, *et al.* The Ewing's family of tumors—a subgroup of small-round-cell tumors defined by specific chimeric transcripts. *N Engl J Med* 1994; 331:294–9.
17 Scotlandi K, Serra M, Manara MC, *et al.* Immunostaining of the p30/32MIC2 antigen and molecular detection of EWS rearrangements for the diagnosis of Ewing's sarcoma and peripheral neuroectodermal tumor. *Hum Pathol* 1996; 27:408–16.
18 Zoubek A, Dockhorn Dworniczak B, Delattre O, *et al.* Does expression of different EWS chimeric transcripts define clinically distinct risk groups of Ewing's tumor patients? *J Clin Oncol* 1996; 14:1245–51.
19 Jeon IS, Davis JN, Braun BS, *et al.* A variant Ewing's sarcoma translocation (7;22) fuses the EWS gene to the ETS gene ETV1. *Oncogene* 1995; 10:1229–34.
20 Kaneko Y, Yoshida K, Handa M, *et al.* Fusion of an ETS-family gene, E1AF, to EWS by t(17;22)(q12;q12) chromosome translocation in an undifferentiated sarcoma of infancy. *Genes Chromosomes Cancer* 1996; 15:115–21.
21 Peter M, Couturier J, Pacquement H, *et al.* A new member of the ETS family fused to EWS in Ewing tumors. *Oncogene* l997; 14:1159–64.
22 Sorensen PH, Lessnick SL, Lopez Terrada D, *et al.* A second Ewing's sarcoma translocation, t(21;22), fuses the EWS gene to another ETS-family transcription factor, ERG. *Nat Genet* 1994; 6:146–51.
23 Urano F, Umezawa A, Hong W, *et al.* A novel chimera gene between EWS and E1A-F, encoding the adenovirus E1A enhancer-binding protein, in extraosseous Ewing's sarcoma. *Biochem Biophys Res Commun* 1996; 219:608–12.
24 Zucman J, Melot T, Desmaze C, *et al.* Combinatorial generation of variable fusion proteins in the Ewing's family of tumours. *EMBO J* 1993; 12:4481–7.
25 Ladanyi M, Lewis R, Garin-Chesa P, *et al.* EWS rearrangement in Ewing's sarcoma and peripheral neuroectodermal tumor. Molecular detection and correlation with cytogenetic analysis and MIC2 expression. *Diagn Mol Pathol* 1993; 2:141–6.
26 Sorensen PH, Shimada H, Liu XF, *et al.* Biphenotypic sarcomas with myogenic and neural differentiation express the Ewing's sarcoma EWS/FLI1 fusion gene. *Cancer Res* 1995; 55:1385–92.
27 Thorner P, Squire J, Chilton MacNeil S, *et al.* Is the EWS/FLI-1 fusion transcript specific for Ewing's sarcoma and peripheral primitive neuroectodermal tumor? A report of four cases showing this transcript in a wider range of tumor types. *Am J Pathol* 1996; 148:1125–38.
28 Sorensen PH, Wu JK, Berean KW, *et al.* Olfactory neuroblastoma is a peripheral primitive neuroectodermal tumor related to Ewing's sarcoma. *Proc Natl Acad Sci USA* 1996; 93:1038–43.
29 Devaney K, Wenig BM, Abbondanzo SL. Olifactory neuroblastoma and other round cell lesions of the sinonasal region. *Mod Pathol* 1996; 9:658–63.
30 Burchill SA, Wheeldon J, Cullinane C, *et al.* EWS–FLI1 fusion transcripts identified in patients with typical neuroblastoma. *Eur J Cancer* 1997; 33:239–43.
31 Desmaze C, Zucman J, Delattre O, *et al.* Interphase molecular cytogenetics of Ewing's sarcoma and peripheral neuroepithelioma t(11;22) with flanking and overlapping cosmid probes. *Cancer Genet Cytogenet* 1994; 74:13–8.
32 Desmaze C, Zucman J, Delattre O, *et al.* Unicolor and bicolor in situ hybridization in the diagnosis of peripheral neuroepithelioma and related tumors. *Genes Chromosomes Cancer* 1992; 5:30–4.
33 Crozat A, Aman P, Mandahl N, *et al.* Fusion of CHOP to a novel RNA-binding protein in human myxoid liposarcoma. *Nature* 1993; 363:640–4.
34 Rabbitts TH, Forster A, Larson R, *et al.* Fusion of the dominant negative transcription regulator CHOP with a novel gene FUS by translocation t(12;16) in malignant liposarcoma. *Nat Genet* 1993; 4:175–80.
35 Zinszner H, Albalat R, Ron D. A novel effector domain from the RNA-binding protein TLS or EWS is required for oncogenic transformation by CHOP. *Genes Dev* 1994; 8:2513–26.
36 Bertolotti A, Lutz Y, Heard DJ, *et al.* hTAF(II)68, a novel RNA/ssDNA-binding protein with homology to the pro-oncoproteins TLS/FUS and EWS is associated with both TFIID and RNA polymerase II. *EMBO J* 1996; 15:5022–31.
37 Hackl W, Luhrmann R. Molecular cloning and subcellular localisation of the snRNP associated protein 69KD, a structural homologue of the proto-oncoproteins TLS and EWS with RNA and DNA-binding properties. *JMol Biol* 1996; 264:843–51.
38 Alliegro MC, Alliegro MA. A nuclear protein regulated during the transition from active to quiescent phenotype in cultured endothelial cells. *Dev Biol* 1996; 174:288–97.
39 Immanuel D, Zinszner H, Ron D. Association of SARFH (sarcoma-associated RNA-binding fly homolog) with regions of chromatin transcribed by RNA polymerase II. *Mol Cell Biol* 1995; 15:4562–71.
40 Stolow DT, Haynes SR. Cabeza, a *Drosophila* gene encoding a novel RNA binding protein, shares homology with EWS and TLS, two genes involved in human sarcoma formation. *Nucleic Acids Res* 1995; 23:835–43.
41 Burd CG, Dreyfuss G. Conserved structures and diversity of functions of RNA-binding proteins. *Science* 1994; 265:615–21.
42 Courey AJ, Tjian R. Analysis of Sp1 in vivo reveals multiple transcriptional domains, including a novel glutamine-rich activation motif. *Cell* 1988; 55:887–98.
43 Plougastel B, Zucman J, Peter M, *et al.* Genomic structure of the EWS gene and its relationship to EWSR1, a site of tumor-associated chromosome translocation. *Genomics* 1993; 18:609–15.

44 Delattre O, Zucman J, Plougastel B, *et al.* Gene fusion with an ETS DNA-binding domain caused by chromosome translocation in human tumours. *Nature* 1992; 359:162–5.
45 Calvio C, Neubauer G, Mann M, *et al.* Identification of hnRNP P2 as TLS/FUS using electrospray mass spectrometry. *RNA* 1995; 1:724–33
46 Alliegro MC, Alliegro MA Identification of a new coiled body component. *Exp Cell Res* 1996; 227:386–90.
47 Zinszner H, Immanuel D, Yin Y, *et al.* A topogenic role for the oncogenic N-terminus of TLS: nucleolar localization when transcription is inhibited. *Oncogene* 1997; 14:451–61.
48 Panagopoulos I, Hxglund M, Mertens F, *et al.* Fusion of the EWS and CHOP genes in myxoid liposarcoma. *Oncogene* 1996; 12:489–94.
49 Ichikawa H, Shimizu K, Hayashi Y, *et al.* An RNA-binding protein gene, TLS/FUS, is fused to ERG in human myeloid leukemia with t(16;21) chromosomal translocation. *Cancer Res* 1994; 54:2865–8.
50 Panagopoulos I, Aman P, Fioretos T, *et al.* Fusion of the FUS gene with ERG in acute myeloid leukemia with t(16;21)(p11;q22). *Genes Chromosomes Cancer* 1994; 11:256–62.
51 Kong XT, Ida K, Ichikawa H, *et al.* Consistent detection of TLS/FUS-ERG chimeric transcripts in acute myeloid leukemia with t(16;21)(p11;q22) and identification of a novel transcript. *Blood* 1997; 90:1192–9.
52 Clark J, Benjamin H, Gill S, *et al.* Fusion of the EWS gene to CHN, a member of the steroid/thyroid receptor gene superfamily, in a human myxoid chondrosarcoma. *Oncogene* 1996; 12:229–35.
53 Ladanyi M, Gerald W. Fusion of the EWS and WT1 genes in the desmoplastic small round cell tumor. *Cancer Res* 1994; 54:2837–40.
54 Zucman J, Delattre O, Desmaze C, *et al.* EWS and ATF-1 gene fusion induced by t(12;22) translocation in malignant melanoma of soft parts. *Nat Genet* 1993; 4:341–5.
55 Labelle Y, Zucman J, Stenman G, *et al.* Oncogenic conversion of a novel orphan nuclear receptor by chromosome translocation. *Hum Mol Genet* 1995; 4:2219–26.
56 Zucman Rossi J, Batzer MA, Stoneking M, *et al.* Interethnic polymorphism of EWS intron 6: genome plasticity mediated by Alu retroposition and recombination. *Hum Genet* 1997; 99:357–63.
57 Rudiger NS, Gregersen N, Kielland Brandt MC. One short well conserved region of Alu-sequences is involved in human gene rearrangements and has homology with prokaryotic chi. *Nucleic Acids Res* 1995; 23:256–60.
58 Parkin DM, Stiller CA, Nectoux J. International variations in the incidence of childhood bone tumours. *Int J Cancer* 1993; 53:371–6.
59 Stiller CA, Parkin DM. Geographic and ethnic variations in the incidence of childhood cancer. *Br Med Bull* 1996; 52:682–703.
60 Bhagirath T, Abe S, Nojima T, *et al.* Molecular analysis of a t(11;22) translocation junction in a case of Ewing's sarcoma. *Genes Chromosomes Cancer* 1995; 13:126–32.
61 Liu J, Nau MM, Zucman Rossi J, *et al.* LINE-I element insertion at the t(11;22) translocation breakpoint of a desmoplastic small round cell tumor. *Genes Chromosomes Cancer* 1997; 18:232–9.
62 Peter M, Mugneret F, Aurias A, *et al.* An EWS/ERG fusion with a truncated N-terminal domain of EWS in a Ewing's tumor. *Int J Cancer* 1996; 67:339–42.
63 Kaneko Y, Kobayashi H, Handa M, *et al.* EWS–ERG fusion transcript produced by chromosomal insertion in a Ewing's sarcoma. . *Genes Chromosomes Cancer* 1997; 18:228–31.
64 Selleri L, Giovannini M, Romo A, *et al* Cloning of the entire FLI1 gene, disrupted by the Ewing's sarcoma translocation breakpoint on 11q24, in a yeast artificial chromosome. *Cytogenet Cell Genet* 1994; 67:129–36.
65 Zucman Rossi J, Legoix P, Thomas G. Identification of new members of the Gas2 and Ras families in the 22ql2 chromosome region *Genomics* 1996; 38:247–54.
66 Meyer D, Wolff CM, Stiegler P, *et al.* Xl-fli, the *Xenopus* homologue of the fli-1 gene, is expressed during embryogenesis in a restricted pattern evocative of neural crest cell distribution. *Mech Dev* 1993; 44:109–21
67 Bailly RA, Bosselut R, Zucman J, *et al.* DNA-binding and transcriptional activation properties of the EWS–FLI-1 fusion protein resulting from the t(11;22) translocation in Ewing's sarcoma. *Mol Cell Biol* 1994; 14:3230–41.
68 Ohno T, Rao VN, Reddy ES. EWS/Fli-1 chimeric protein is a transcriptional activator. *Cancer Res* 1993; 53:5859–63.
69 Prasad DD, Ouchida M, Lee L, *et al.* TLS/FU fusion domain of TLS/FUS-erg chimeric protein resulting from the t(16;21) chromosomal translocation in human myeloid leukemia functions as a transcriptional activation domain. *Oncogene* 1994; 9:3717–29.
70 Donaldson LW, Petersen JM, Graves BJ, *et al.* Solution structure of the ETS domain from murine Ets-1:a winged helix-turn-helix DNA binding motif. *EMBO J* 1996; 15:125–34.
71 Liang H, Olejniczak ET, Mao X, *et al.* The secondary structure of the ets domain of human Fli-1 resembles that of the helix-turn-helix DNA-binding motif of the *Escherichia coli* catabolite gene activator protein. *Proc Natl Acad Sci USA* 1994; 91:11655–9
72 Liang H, Mao X, Olejniczak ET, *et al.* Solution structure of the ets domain of Fli-1 when bound to DNA. *Nat Struct Biol* 1994; 1:871–5.
73 Jonsen MD, Petersen JM, Xu QP, *et al.* Characterization of the cooperative function of inhibitory sequences in Ets-1. *Mol Cell Biol* 1996; 16:2065–73.
74 Petersen JM, Skalicky JJ, Donaldson LW, *et al.* Modulation of transcription factor Ets-1 DNA binding: DNA-induced unfolding of an alpha helix. *Science* 1995; 269:1866–9.
75 Wasylyk B, Hahn SL, Giovane A. The Ets family of transcription factors [published erratum appears In *Eur J Biochem* 1993 Aug 1;215(3):907]. *Eur J Biochem* 1993; 211:7–18.
76 Werner MH, Clore M, Fisher CL, *et al.* The solution structure of the human ETS1-DNA complex reveals a novel mode of binding and true side chain intercalation. *Cell* 1995; 83:761–71.
77 Macleod K, Leprince D, Stehelin D. The ets gene family. *Trends Biochem Sci* 1992; 17:251–6.
78 Fitzsimmons D, Hodsdon W, Wheat W, *et al.* Pax-5 (BSAP) recruits Ets proto-oncogene family proteins to form functional ternary complexes on a B-cell-specific promoter. *Genes Dev* 1996; 10:2198–211.
79 Jousset C, Carron C, Boureux A, *et al.* A domain of TEL conserved in a subset of ETS proteins defines a specific oligomerization interface essential to the mitogenic properties of the TEL-PDGFR beta oncoprotein. *EMBO J* 1997; 16:69–82.
80 Braun BS, Frieden R, Lessnick SL, *et al.* Identification of target genes for the Ewing's sarcoma EWS/FLI fusion protein by representational difference analysis. *Mol Cell Biol* 1995; 15:4623–30.

81 Thompson AD, Braun BS, Arvand A, *et al.* EAT-2 is a novel SH2 domain containing protein that is up regulated by Ewing's sarcoma EWS/FLI1 fusion gene. *Oncogene* 1996; 13:2649–58.
82 Kaya M, Yoshida K, Higashino F, *et al.* A single ets-related transcription factor, E1AF, confers invasive phenotype on human cancer cells. *Oncogene* 1996; 12:221–7.
83 Hida K, Shindoh M, Yasuda M, *et al.* Antisense E1AF transfection restrains oral cancer invasion by reducing matrix metalloproteinase activities. *Am J Pathol* 1997; 150:2125–32.
84 Wasylyk C, Gutman A, Nicholson R, *et al.* The c-Ets oncoprotein activates the stromelysin promoter through the same elements as several non-nuclear oncoproteins. *EMBO J* 1991; 10:1127–34.
85 Buttice G, Duterque Coquillaud M, Basuyaux JP, *et al.* Erg, an Ets-family member, differentially regulates human collagenase1 (MMP1) and stromelysin1 (MMP3) gene expression by physically interacting with the Fos/Jun complex. *Oncogene* 1996; 13:2297–306.
86 Kovar H, Aryee DN, Jug G, *et al.* EWS/FLI-1 antagonists induce growth inhibition of Ewing's tumor cells in vitro. *Cell Growth Differ* 1996; 7:429–37.
87 Pingoud V, Zinck R, Hipskind RA, *et al.* Heterogeneity of ternary complex factors in HeLa cell nuclear extracts. *J Biol Chem* 1994; 269:23310–7.
88 Magnaghi Jaulin L, Masutani H, Robin P, *et al.* SRE elements are binding sites for the fusion protein EWS–FLI-1. *Nucleic Acids Res* 1996; 24:1052–8.
89 Watson DK, Robinson L, Hodge DR, *et al.* FLI1 and EWS–FLI1 function as ternary complex factors and ELK1 and SAP1a function as ternary and quaternary complex factors on the Egr1 promoter serum response elements *Oncogene* 1997; 14:213–21.
90 Cavazzana AO, Miser JS, Jefferson J, *et al.* Experimental evidence for a neural origin of Ewing's sarcoma of bone. *Am J Pathol* 1987; 127:507–18.
91 Ben David Y, Giddens EB, Letwin K, *et al.* Erythroleukemia induction by Friend murine leukemia virus: insertional activation of a new member of the ets gene family, Fli-1, closely linked to c-ets-1. *Genes Dev* 1991; 5:908–18.
92 Howard JC, Yousefi S, Cheong G, *et al.* Temporal order and functional analysis of mutations within the Fli-1 and p53 genes during the erythroleukemias induced by F-MuLV. *Oncogene* 1993; 8:2721–9
93 Hart AH, Corrick CM, Tymms MJ, *et al.* Human ERG is a proto-oncogene with mitogenic and transforming activity *Oncogene* 1995; 10:1423–30
94 Lessnick SL, Braun BS, Denny CT, *et al.* Multiple domains mediate transformation by the Ewing's sarcoma EWS/FLI-1 fusion gene. *Oncogene* 1995; 10:423–31.
95 May WA, Gishizky ML, Lessnick SL, *et al.* Ewing's sarcoma 11;22 translocation produces a chimeric transcription factor that requires the DNA-binding domain encoded by FLI1 for transformation. *Proc Natl Acad Sci USA* 1993; 90:5752–6
96 Toretsky JA, Connell Y, Neckers L, *et al* Inhibition of EWS–FLI-1 fusion protein with antisense oligodeoxynucleotides. *J Neurooncol* 1997; 31:9–16.
97 Tanaka K, Iwakuma T, Harimaya K, *et al.* EWS-Fli1 antisense oligodeoxynucleotide inhibits proliferation of human Ewing's sarcoma and primitive neuroectodermal tumor cells. *J Clin Invest* 1997; 99:239–47.
98 Ouchida M, Ohno T, Fujimura Y, *et al.* Loss of tumorigenicity of Ewing's sarcoma cells expressing antisense RNA to EWS-fusion transcripts. *Oncogene* 1995; 11:1049–54
99 Toretsky JA, Kalebic T, Blakesley V, *et al.* The insulin-like growth factor-1 receptor is required for EWS/FLI1 transformation of fibroblasts. *J Biol Chem* 1997; 272:30822–7.
100 Hofbauer S, Hamilton G, Theyer G, *et al.* Insulin-like growth factor-I-dependent growth and in vitro chemosensitivity of Ewing's sarcoma and peripheral primitive neuroectodermal tumour cell lines. *Eur J Cancer* 1993; 29A:241–5.
101 van Valen F, Winkelmann W, Jurgens H. Type I and type II insulin-like growth factor receptors and their function in human Ewing's sarcoma cells. *J Cancer Res Clin Oncol* 1992; 118:269–75.
102 Yee D, Favoni RE, Lebovic GS, *et al.* Insulin-like growth factor I expression by tumors of neuroectodermal origin with the t(11;22) chromosomal translocation. A potential autocrine growth factor. *J Clin Invest* 1990; 86:1806–14.
103 Scotlandi K, Benini S, Sarti M, *et al.* Insulin-like growth factor I receptor-mediated circuit in Ewing's sarcoma/peripheral neuroectodermal tumor: a possible therapeutic target. *Cancer Res* 1996; 56:4570–4.
104 Baserga R, Hongo A, Rubini M, *et al.* The IGF-I receptor in cell growth, transformation and apoptosis. *Biochim Biophys Acta* 1997; 1332:F105–26.
105 Yi H, Fujimura Y, Ouchida M, *et al.* Inhibition of apoptosis by normal and aberrant Fli-1 and erg proteins involved in human solid tumors and leukemias. *Oncogene* 1997; 14:1259–68.
106 Mugneret F, Lizard S, Aurias A, *et al.* Chromosomes in Ewing's sarcoma. II. Nonrandom additional changes, trisomy 8 and der(16)t(1;16). *Cancer Genet Cytogenet* 1988; 32:239–45.
107 Armengol G, Tarkkanen M, Virolainen M, *et al.* Recurrent gains of 1q, 8 and 12 in the Ewing's family of tumours by comparative genomic hybridization. *Br.J Cancer* 1997; 75:1403–9.
108 Jost CA, Marin MC, Kaelin WG, Jr. p73 is a human p53-related protein that can induce apoptosis. *Nature* 1997; 389:191–4.
109 Kaghad M, Bonnet H, Yang A, *et al.* Monoallelically expressed gene related to p53 at 1p36, a region frequently deleted in neuroblastoma and other human cancers. *Cell* 1997; 90:809–19.
110 Kovar H, Auinger A, Jug G, *et al.* Narrow spectrum of infrequent p53 mutations and absence of MDM2 amplification in Ewing's tumours. *Oncogene* 1993; 8:2683–90.
111 Ladanyi M, Lewis R, Jhanwar SC, *et al.* MDM2 and CDK4 gene amplification in Ewing's sarcoma. *J Pathol* 1995; 175:211–7.
112 Aryee DN, Strobel T, Kos K, *et al.* High nm23-H1/NDPK-A expression in Ewing's tumors: paradoxical immunohistochemical reactivity and lack of prognostic significance. *Int J Cancer* 1995; 64:104–11.
113 Kovar H, Jug G, Aryee DNT, *et al.* Among genes involved in the RB dependent cell cycle regulatory cascade, the p16 tumor suppressor gene is frequently lost in the Ewing's family of tumors. *Oncogene* 1997; 15:2225–32.
114 MacLachlan TK, Sang N, Giordano A. Cyclins, cyclin-dependent kinases and cdk inhibitors: implications in cell cycle control and cancer. *Crit Rev Eukaryot Gene Expr* 1995; 5:127–56.
115 Hamelin R, Zucman J, Melot T, *et al* p53 mutations in human tumors with chimeric EWS/FLI-l genes. *Int J Cancer* 1994; 57:336–40.
116 Kovar H, Jug G, Gadner H. Variable proportion of tumor cells refractory to p53 mediated apoptosis: a mechanism of therapy resistance in Ewing's tumors? *Med Ped Oncol* 1997; 29:327

117 Prasad SC, Thraves PJ, Bhatia KG, *et al.* Enhanced poly(adenosine diphosphate ribose) polymerase activity and gene expression in Ewing's sarcoma cells. *Cancer Res* 1990; 50:38–43.
118 Peter M, Magdelenat H, Michon J, *et al.* Sensitive detection of occult Ewing's cells by the reverse transcriptase-polymerase chain reaction. *Br J Cancer* 1995; 72:96–100.
119 Pfleiderer C, Zoubek A, Gruber B, *et al.* Deflection of tumour cells in peripheral blood and bone marrow from Ewing's tumour patients by RT-PCR. *Int J Cancer* 1995; 64:135–9.
120 Zoubek A, Kovar H, Kronberger M, *et al.* Mobilization of tumour cells during biopsy in an infant with Ewing's sarcoma. *Eur J Pediatr* 1996; 155:373–6.
121 Zoubek A, Ladenstein R, Windhager R, *et al.* Predictive potential of testing for bone marrow involvement in Ewing's tumor patients by RT-PCR: a preliminary evaluation. *Int J Cancer* 1998; 79:56–60.
122 Tanaka K, Iwakuma T, Harimaya K, *et al.* EWS–Fli1 antisense oligodeoxynucleotide inhibits proliferation of human Ewing's sarcoma and primitive neuroectodermal tumor cells. *J Clin Invest* 1997; 99:239–47.
123 Mao X, Miesfeldt S, Yang H, *et al.* The FLI-1 and chimeric EWS–FLI-1 oncoproteins display similar DNA binding specificities. *J Biol Chem* 1994; 269:18216–22.
124 Zoubek A, Pfleiderer C, Ambros PF, *et al.* Minimal metastatic and minimal residual disease in patients with Ewing's tumors. *Klin Pädiatr* 1995; 207:242–7.
125 Petermann R, Mossier B, Aryee DNT, *et al.* Oncogenic EWS-FLI1 interacts with hsRPB7, a subunit of human RNA polymerase II. *Oncogene* 1998; in press.
126 Bertolotti A, Melot T, Acker J, *et al.* EWS, but not EWS-Fli1, is associated with both TFIID and RNA polymerase II: Interactions between two members of the TET family, EWS and hTAFII68, and subunits of TFIID and RNA polymerase II subcomplexes. *Mol Cell Biol* 1998; 18:1489–97.
127 Aryee DNT, Petermann R, Kos K, *et al.* Cloning of a novel human ELF-1-related ETS transcription factor, ELFR, its characterization and chromosomal assignment relative to ELF-1. *Gene* 1998; 210:71–8.
128 De Alava E, Kawai A, Healey JA, *et al.* EWS-FLI1 fusion transcript structure is an independent determinant of prognosis in Ewing's sarcoma. *J Clin Oncol* 1998; 16:1248–55.
